# Effect of microbial fertilizers on soil microbial community structure in rotating and continuous cropping *Glycyrrhiza uralensis*

**DOI:** 10.3389/fpls.2024.1452090

**Published:** 2025-01-07

**Authors:** Daiyu Qiu, Xue Wang, Kan Jiang, Gaoxia Gong, Fang Bao

**Affiliations:** ^1^ College of Agronomy, Gansu Agricultural University, Gansu, China; ^2^ Gansu Key Laboratory of Arid Habitat Crop Science, Gansu Agricultural University, Gansu, China; ^3^ Science and Technology R&D Department, China Chinese Medicine Co., LTD, Beijing, China

**Keywords:** microbial fertilizers, *Glycyrrhiza uralensis*, crop rotation, continuous cropping, soil microorganism

## Abstract

**Introduction:**

*Glycyrrhiza uralensis* is a perennial medicinal plant. It’s generally cultivated for three years, and should avoid long-term continuous cultivation. However, unreasonable crop rotation and extensive fertilization are common in *G. uralensis* cultivation, which leads to the imbalance of soil microflora structure, and the obstacle of continuous cropping are becoming increasingly serious. Some microbial fertilizers such as *Bacillus amyloliquefaciens, Bacillus subtilis*, and complex microbial agent have the advantage of regulating soil microbial community structure and improving the soil environment. Therefore, these three kinds of microbial fertilizers were applied to *G. uralensis* and their effects on soil microorganisms of *G. uralensis* were studied.

**Methods:**

Combine microbial fertilizers with conventional fertilization for continuous cropping and rotating *G. uralensis*. High-throughput sequencing technology was used to determine soil microbial richness, diversity and distribution of community structure in rotating and continuous cropping *G. uralensis*.

**Results and discussion:**

Continuous cropping reduced *G. uralensis* soil bacterial diversity by 7.56% and increased fungal richness by 17.01% compared with crop rotation. However, after the application of microbial fertilizers, the fungal richness and diversity of continuous cropping *G. uralensis* were significantly reduced by 4.76%~20.96%, and the soil bacterial diversity of continuous cropping and rotating *G. uralensis* was significantly increased by 7.22%~12.03% and 6.75%~11.69% compared with the respective controls, respectively. Continuous cropping and rotating *G. uralensis* soil dominant bacteria mainly include *Proteobacteria, Actinobacteria* and *Gemmatimonadota*, and the dominant fungi include *Ascomycota, Basidiomycota* and *Zygomycota*. The activity process of these microbial communities was mainly through carbohydrate metabolism and amino acid synthesis pathway in metabolism. The complex microbial agent significantly increased the relative abundance of soil dominant bacteria communities of continuous cropping and rotating *G. uralensis* by 3.11~11.54 percentage points, and significantly reduced the relative abundance of soil dominant fungal communities of continuous cropping *G. uralensis* by 1.57~8.93 percentage points, compared with the control. Of the three microbial fertilizers, the complex microbial agent had the most significant effects on optimizing the soil microbial community structure of continuous cropping and rotating *G. uralensis*. Conclusion: the application effect of different microbial fertilizers in continuous cropping *G. uralensis* was better than crop rotation, and the application effect of complex microbial agent was the best, which has more application value and development prospect in the cultivation management of *G. uralensis*.

## 1 Introduction

*Glycyrrhiza uralensis* Fisch is a perennial root plant of Leguminosae *Glycyrrhiza* L., with root and root stem as medicine, is also widely used in food, health care products, daily chemical industry, animal husbandry and other industries (NPC., 2020). *G. uralensis* has the characteristics of drought and cold resistance, salt and alkali resistance, and not easy to be covered by sand. It is an excellent plant for windbreak and sand fixation and improved salt-alkali soil in the desert and semi-desert area of northwest China. It has both economic and ecological value (Ji et al., 2024). Nowadays, the reserves of wild *G. uralensis* resources are decrease sharply, but the demand for *G. uralensis* is increasing year by year. The wild *G. uralensis* resources distributed in China have been unable to meet the increasing market demand, and the market contradiction of *G. uralensis* resources in short supply is becoming increasingly prominent (Yu et al., 2022). This situation promotes the continuous expansion of the cultivation area of *G. uralensis* in China, and the *G. uralensis* cultivated in various producing areas has developed into the main supply source of medicinal *G. uralensis* (Zhi et al., 2022). However, the traditional *G. uralensis* cultivation technology and management process are relatively extensive, and there are some problems in the planting process, such as not paying attention to reasonable crop rotation and blind application of chemical fertilizers (Ting et al., 2023). These problems lead to the imbalance of soil microbial community structure, the frequent occurrence of soil-borne diseases, and the decline of soil integrated environment, which seriously restricts the green and sustainable development of *G. uralensis* cultivation (Xin et al., 2022).

In plant-soil-microbe systems, one side, functional strains in microbial fertilizers can modulate signaling pathways, increase gene expression, promote proline synthesis, and inhibit the growth of pathogens (Chaudhary et al., 2022). On the other side, the beneficial strains in microbial fertilizers can form a dominant bacterial community locally in the soil, guide the development of environmental microbiota to a benign direction, so as to facilitate soil organic matter decomposition and nutrient transformation, enhance the absorption of nutrients with plants, and promote plant growth (Zaeem et al., 2019). On the contrary, the pores of soil particles provide contact points for microbial colonization, and the available nutrients such as soil nitrogen, phosphorus and potassium improve the nutrients for soil microorganisms, ensuring the reproduction and growth of microorganisms in microbial fertilizers (Vieira et al., 2020; Deepak et al., 2016).

At present, the rational application of microbial fertilizer has been proved to be an effective measure to balance the structure of crop soil microflora, enhance crop disease resistance, and improve crop yield and quality (Plocek et al., 2024). For instance, foliar sprays of different microbial fertilizers have a positive effect on the growth of *Glycine max* (L.) Merr. and *Zea mays* L., including increased enzyme activity, metabolites, nutrient concentration, mitigated effects of salinity, and increased biomass (Vela et al., 2018; Guimaraes et al., 2020). Chinese cabbage with different *Trichoderma* fungi applied via irrigation resulted in a significant increase in cabbage yield (by 37%) and enhanced enzyme activity in the soil (Ji et al., 2020). In addition, various strains of *Bacillus subtilis*, *Bacillus megaterium*, and *Bacillus pumilus* have resulted in rate of growth increases, stress tolerances, and nutrient metabolites in lettuce and tomatoes. These studies provide the theoretical basis and application ideas for the application of microbial fertilizers in the planting process of *G. uralensis*.

However, the research and application of microbial fertilizers mainly focus on vegetables, *Triticum aestivum* L., *Zea mays* L., and other crops, its influence on the soil microbiological environment of medicinal plants such as *G. uralensis* is rarely systematically reported (Bao et al., 2024). Therefore, in this study, different types of microbial fertilizers were applied in continuous cropping and rotating *G. uralensis* based on conventional fertilization. By analyzing the results of high-throughput sequencing, this study compared the microbial richness, diversity, community structure and metabolic pathways of continuous cropping and rotating *G. uralensis*. Explored the influence of different microbial fertilizers on the soil microorganisms of rotating and continuous cropping *G. uralensis*, and screened the microbial fertilizers with the best application effect. It is expected to provide effective prevention and control measures to alleviate the obstacles of *G. uralensis* and provide theoretical basis for the application of microbial fertilizers in the cultivation of *G. uralensis*.

## 2 Materials and methods

### 2.1 Experimental site and basic agrochemical characteristics of soil

The experimental site is located in Changning Town, Minqin County, Gansu Province, China (102° 24′ 55″ E, 38° 53′ 31″ N, elevation of 1 302.0 m). This area belongs to the temperate continental arid climate area, with an annual average temperature of 8.5°C, annual average precipitation of 113.2 mm, average evaporation of 2 412 mm, frost-free period of 162 d. The soil is sandy loam soil, irrigation is convenient, suitable for planting *G. uralensis* and other rhizome medicinal materials.

In 2019 and 2020, *G. uralensis* was planted in the continuous experimental fields, and on July 30, 2021, the continuous cropping *G. uralensis* was harvested. From May to October 2020, *Zea mays* L. was planted in crop rotation experimental fields, from May to July 2021, *Citrullus lanatus* was planted here, and on July 30, 2021, *Citrullus lanatus* was harvested. The soil base fertility (0~20 cm soil layer) in the continuous cropping experimental site was soil organic matter 17.06 g/kg, total nitrogen 12.49 g/kg, total phosphorus 0.29 g/kg, total potassium 5.05 g/kg, ammonium nitrogen 18.97 mg/kg, available phosphorus 16.45 mg/kg, available potassium 142.54 mg/kg. The soil base fertility in the rotation experimental site was soil organic matter 21.12 g/kg, total nitrogen 14.88 g/kg, total phosphorus 0.33 g/kg, total potassium 5.71 g/kg, ammonium nitrogen 22.16 mg/kg, available phosphorus 11.21 mg/kg, available potassium 113.04 mg/kg.

### 2.2 Experimental materials

*G. uralensis* seed: provided by China Pharmaceutical Seed Co., LTD., was identified as the *G. uralensis* Fisch seed by associate professor Qiu Daiyu of Gansu Agricultural University in Gansu, China.

Microbial fertilizers: *Bacillus amyloliquefaciens* (powder, the effective number of viable bacteria exceeds 1×10^10^/g), and the main species are *Bacillus amyloliquefaciens* and its metabolites. *Bacillus subtilis* (granules, the effective number of viable bacteria exceeds 1×10^10^/g), and the main species are *Bacillus subtilis* and its metabolites. Both fertilizers were produced by Jiangxi Weizhixing Biological Technology Co., LTD (Jiangxi, China). Complex microbial agent (liquid, the effective number of viable bacteria exceeds 1×10^10^/mL), and the main strains are photosynthetic bacteria, lactic acid bacteria, yeast and three major bacterial groups of multiple microorganisms. It was produced by Sinochem Agricultural Ecological Science and Technology Co., LTD (Hubei province, China).

Chemical fertilizers: Diammonium phosphate is a granule (N~P_2_O_5_~K_2_O: 18~46~0, total nutrient exceeds 64.0%), which was produced by Yunnan Xiangfeng Chemical Fertilizer Co., LTD (Yunnan province, China). Compound fertilizer is also a granule (N~P_2_O_5_~K_2_O: 15~15~15), which was produced by Fujian Sinochem Zhisheng Chemical Fertilizer Co., LTD (Fujian province, China).

### 2.3 Experimental design

In the experiment, split block design was adopted, and the primary area has two kinds of crop rotation and continuous cropping. Four fertilization treatments in the secondary area, namely conventional fertilization (CK), *Bacillus amyloliquefaciens* (J), *Bacillus subtilis* (K) and complex microbial agent (F). Three replicates of each treatment, with a total of 24 plots, and the plot area is 5 m ×3 m, and a 1 m-wide protection line was set around the plot.

On August 3, 2021, *G. uralensis* was sown in continuous cropping and rotation experiment site, and plant spacing 15 cm, line spacing 25 cm, sowing depth 2.5~3.0 cm, 5~7 grains per hole, and after *G. uralensis* seedling emergence, each hole was reserved with 2 seedlings. Before sowing, the base fertilizer (diammonium phosphate, compound fertilizer) and microbial fertilizers were applied in combination with rotary tillage. Among them, *Bacillus amyloliquefaciens* and *Bacillus subtilis* were mixed with base fertilizers and applied at one time. The complex microbial agent is liquid, the stock solution is diluted to 800 times, and applied when irrigation. The amount of fertilization is shown in **Table 1**. In the second and third year, after the *G. uralensis* seedlings grow, and on May 20, July 20 and September 20, for the crop rotation and continuous cropping *G. uralensis* irrigation. Before irrigation, the control group applied topdressing fertilizer, and the treatment groups applied both topdressing fertilizer and microbial fertilizers simultaneously, and the amount of fertilization was the same as that of base fertilizers and microbial fertilizers during sowing.

**Table 1:** Fertilization amount under different treatments in experimental plots of rotating and continuous cropping *G. uralensis*.

Crops for rotation	Treatment	Crop rotation/Continuous cropping				
		Base fertilizer/Top dressing		Microbial fertilizers		
		Compound fertilizer kg/ha	Diammonium phosphate kg/ha	*Bacillus amyloliquefaciens* kg/ha	*Bacillus subtilis* kg/ha	Complex microbial agent mL/m^2^
Crop rotation	CK	375.18	375.18	0	0	0
	J	375.18	375.18	15.00	0	0
	K	375.18	375.18	0	15.00	0
	F	375.18	375.18	0	0	3.00
Continuous cropping	CK	375.18	375.18	0	0	0
	J	375.18	375.18	15.00	0	0
	K	375.18	375.18	0	15.00	0
	F	375.18	375.18	0	0	3.00

CK, Conventional fertilization; J, *Bacillus amyloliquefaciens*; K, *Bacillus subtilis*; F, Complex microbial agent. The same as following.

### 2.4 Soil sample collection

15 July 2021 and 25 May, 28 July, and 28 September 2023, utilizing the five-point sampling method, five *G. uralensis* plants without pests and diseases were randomly selected in each plot, near the *G. uralensis* roots, surface litter and debris were removed. Using a sterile shovel (20 cm long, 15 cm wide), soil samples were collected at depth of 0~30 cm. After mixed the five portions of the *G. uralensis* soil from the same plot thoroughly, the soil was sieved through a 2 mm sieve. Soil samples collected on July 15, 2021, were air-dried, sieved through a 0.15 mm sieve, and stored at room temperature for soil nutrient analysis. Other soil samples were placed in a sterile cryotube, taken back to the laboratory, and stored at -80°C for soil microbial biomass determination.

### 2.5 Soil nutrient and microbiological measurement

#### 2.5.1 Soil nutrient content determination

Soil properties were determined according to *Soil Agricultural Chemistry Analysis* (Bao, 2000). The soil organic matter content was analyzed by potassium dichromate titration. The total nitrogen and ammonium nitrogen were assessed by half trace chemical nitrogen method and 2 mol/L KCl extraction-indiophol blue color method. The total phosphorus and available phosphorus were measured using NaOH melting-molybdenum antimony colorimetry and NaHCO_3_ extraction-molybdenum antimony colorimetry. The total potassium and available potassium were determined by NaOH melting-flame photometry and ammonium acetate extraction-flame photometry.

#### 2.5.2 Analysis of rhizosphere soil microbial richness and diversity

1) Extraction of total DNA from soil samples: extraction was performed according to the MOBIO^®^ Laboratories kit, and total DNA purity and concentration were detected by the Quanti Fluor TM-ST blue fluorescence quantification system and 2% agarose gel electrophoresis.

2) PCR amplification: The universal primers F: 5’-ACTCCTACGGGAGGCAGCA-3’ and R: 5’- GGACTACHVGGGTWTCTAAT-3’ were employed for PCR amplification of the bacterial 16S rRNA gene 16S V3-V4 region, and the universal primers F: 5’- GGAAGTAAAAGTCGTAACAAG G-3’ and R: 5’-GCTGCGTTCTTCATCGATGC-3’ were used for PCR amplification of the fungal ITS gene ITS-V1 region. The amplification procedure was 94°C predenaturation for 5 min for another 30 cycles (94°C was denatured for 30 s, 56°C was annealed for 30 s, and 72°C was extended for 30 s), and extend at 72°C for 7 min.

3) High-throughput sequencing: The purified amplified genes were sequenced on the Illumina PE250 platform. The resulting data were first preprocessed, and then the chimeras and short sequences were removed according to the positive and negative barcode sequence and the primer sequences to obtain the final effective sequence. Cluster analysis of valid sequences using the Qiime platform at the 97% similarity level and taxonomic annotation for each OTU (Operational Taxonomic Units) obtained.

4) Calculate soil microbial richness and diversity indices: *Chao1* index was used to reflect community species richness, and *Shannon* index indicates species diversity.


$$Schao1=S_{obs}+\frac{{\text{n}}_{1}\left({\text{n}}_{1}-1\right)}{2\left(n_{2}+1\right)}$$

Where the predicted number of OTUs is *SChao1*; The number of OTUs actually observed is expressed in *S_obs_*: *n_1_* represents the number of OTUs with only one sequence, and *n_2_* represents the number of OTUs with two sequences (Zhu and Yu, 2022).


$$H_{shanmon}=-\sum_{i=1}^{S}P_{i}\ln P_{i}$$

Where *S* is the total number of microbial species in the sample and *i* represents the *i*th species; The relative abundance of microbial species *i* in a sample is represented by *P_i_*, and its formula is as follows: $P_{i}=n_{i}/\text{N}$ , where N is the total number of individuals of all microbial species in a given sample and *n_i_* is the number of individuals of species *i* (Zhu et al., 2018).

### 2.6 Statistical analysis

One-way analysis of variance (ANOVA) was conducted using SPSS 27.0 software (SPSS Inc., Chicago, IL, USA) with a significance level of difference set at *P*<0.05, and multiple comparisons were carried out using the Duncan method. Cluster analysis used the *Paiseno Gene Cloud* platform (https://www.genescloud.cn/login) with 97% similarity threshold as the standard, and the chart production of the cluster analysis was made using the “Heat Map Dendrogram” tool in the Origin 2021 software (Origin Lab, Northampton, MA, USA). The Venn analysis was executed with the aid of the “Venn Diagram” tool. Additionally, the “vegan” R package (version 3.6.3) was employed to conduct principal coordinate analysis (PCoA) based on the Bray-Curtis distance (The R Foundation for Statistical Computing, Vienna, Austria).

## 3 Results and analysis

### 3.1 Analysis of the sequencing results for soil bacteria and fungi

For each treatment, the coverage of bacteria and fungi in rotating and continuous cropping *G. uralensis* was above 98%. This showed that the sequencing depth of bacteria and fungi can cover the vast majority of bacteria and fungi in the soil, and only a few bacterial and fungal species are undiscovered. The sequencing results could reflect the microbial community structure composition of rotating and continuous cropping *G. uralensis* in the experiment, and the data cuuld be used for subsequent analysis (**Table 2**).

**Table 2:** Bacterial and fungal sequencing coverage of soil samples of rotating and continuous cropping *G. uralensis* under different treatments.

Crops for rotation	Treatment	Bacterial coverage (%)	Fungal coverage (%)
Crop rotation	R	98.49 ± 0.001b	98.14 ± 0.001c
	CK	98.16 ± 0.002b	98.23 ± 0.002c
	J	98.34 ± 0.003b	99.02 ± 0.003a
	K	99.42 ± 0.005a	98.61 ± 0.005b
	F	99.10 ± 0.001a	99.19 ± 0.002a
Continuous cropping	G	99.15 ± 0.003a	99.44 ± 0.002a
	CK	98.66 ± 0.002b	98.97 ± 0.002a
	J	98.43 ± 0.003b	99.19 ± 0.003a
	K	99.29 ± 0.005a	98.29 ± 0.005b
	F	99.19 ± 0.001a	99.31 ± 0.002a

R, Soil sample before planting in rotating *G. uralensis*. G, Soil sample before planting in continuous cropping *G. uralensis*. Different lowercase letters indicate significant difference between rotating and continuous cropping of *G. uralensis*. The same as following.

Under different treatments, a total of 722,142 valid sequences were obtained from rotating *G. uralensis* soil bacterial libraries, and 43,329 optimized sequences were obtained after filtering and optimization. OTU clustering of the optimized sequences based on the 97% similarity level yielded an average of 32,401 bacterial OTU. Rotation of *G. uralensis* soil fungal libraries yielded 750,957 valid sequences, 716,365 optimized sequences, and clustered 3,598 fungal OTU. Among them, the number of bacterial and fungal optimized sequences and OTU number of rotating *G. uralensis* were significantly different between the J, K, F and CK treatment. Continuous cropping *G. uralensis* soil bacterial libraries yielded 742,400 valid sequences, 468,168 optimized sequences, and clustered 33,342 fungal OTU, and its fungal libraries yielded 732,143 valid sequences, 704,599 optimized sequences and 3,054 fungal OTU. Among them, J, K and F treatments significantly reduced the number of soil fungi OTU in continuous cropping *G. uralensis* (**Table 3**).

**Table 3:** Optimized sequences and OTU numbers of soil bacteria and fungi in rotating and continuous cropping *G. uralensis* under different treatments.

Crops for rotation	Treatment	Bacteria		Fungi	
		Sequence number	OTU number (97%)	Sequence number	OTU number (97%)
Crop rotation	R	32728.67 ± 894.72b	2358.33 ± 22.30a	49810.67 ± 1069.55b	278.67 ± 20.02a
	CK	27684.67 ± 317.95d	2026.33 ± 95.55d	44516.33 ± 7464.92c	190.33 ± 21.94e
	J	20409.12 ± 230.83e	2038.00 ± 46.00d	41311.00 ± 8079.80d	247.33 ± 50.29c
	K	33818.67 ± 853.28a	2132.67 ± 95.62c	49726.67 ± 6732.00b	217.33 ± 28.29d
	F	29802.00 ± 469.39c	2245.00 ± 60.56b	53423.67 ± 1625.39a	265.67 ± 29.77b
Continuous cropping	G	29516.67 ± 1926.22c	1975.33 ± 262.39d	44637.00 ± 1226.29d	220.00 ± 43.03a
	CK	35494.33 ± 1301.43a	2671.67 ± 194.03a	47310.00 ± 1116.12b	210.00 ± 30.20b
	J	34576.67 ± 1698.94b	2377.67 ± 154.28b	46041.00 ± 1256.86c	200.67 ± 37.75c
	K	29670.67 ± 1712.70c	2099.00 ± 185.94c	48151.00 ± 1326.34a	176.33 ± 52.20d
	F	26797.67 ± 1350.19d	1990.33 ± 139.38d	48727.33 ± 1857.63a	211.00 ± 89.45b

Used the rRNA gene sequence database and the UNITE fungal molecular identification database as the reference databases, the OTU sequences in rotating and continuous cropping *G. uralensis* soil samples were taxonomic annotated, and the species taxonomic information corresponding to each OTU is shown in **Table 4**. In rotating *G. uralensis* soil, the detected bacteria included 17 phyla, 36 classes, 58 orders, 62 families, 65 genera, 1991 species, and the fungi included 6 phyla, 13 classes, 22 orders, 33 families, 43 genera and 405 species. In continuous cropping *G. uralensis* soil, the detected bacteria included 14 phyla, 29 classes, 56 orders, 65 families, 72 genera, 1700 species; and the fungi included 5 phyla, 12 classes, 21 orders, 30 families, 37 genera, 362 species. Among them, the number of bacteria and fungi of rotating *G. uralensis* is more than that of continuous cropping *G. uralensis*.

**Table 4:** Statistical table of soil bacteria and fungi species in rotating and continuous cropping *G. uralensis*.

Crops for rotation	Soil microorganism	Phylum	Class	Order	Family	Genus	Species
Crop rotation	Bacteria	17	36	58	62	65	1991
	Fungi	6	13	22	33	43	405
Continuous cropping	Bacteria	14	29	56	65	72	1700
	Fungi	5	12	21	30	37	362

**Figure 1:**
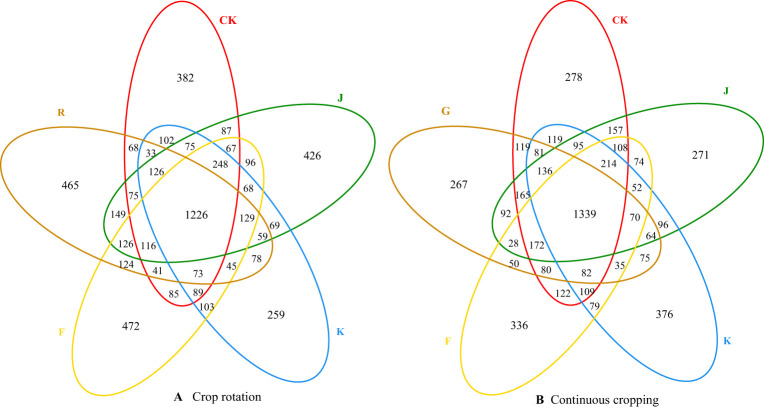
Venn diagram of common and unique OTU numbers of soil bacteria in rotating **(A)** and continuous cropping **(B)** *G. uralensis* with different treatments.

**Figure 2:**
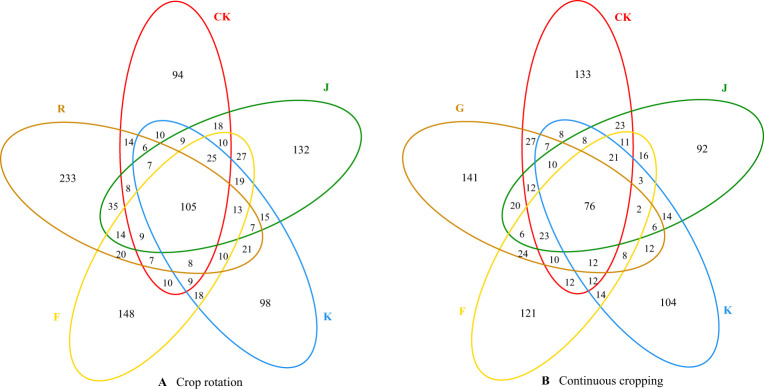
Venn diagram of common and unique OTU numbers of soil fungi in rotating **(A)** and continuous cropping **(B)** *G. uralensis* with different treatments.

The common and unique OTU numbers of soil bacteria and fungi from different treatments are shown in Venn figure (**Figures 1**, **2**). For soil bacteria, the number of common bacterial OTU between soil sample before planting *G. uralensis* in rotating test site (R), CK, J, K and F treatments was 1226, and the number of unique bacterial OTU was 465, 382, 426, 259 and 472, respectively. Among them, J and K treatment significantly increased the number of unique OTU soil bacteria in rotating *G. uralensis*. The number of common bacterial OTU between soil sample before planting *G. uralensis* in continuous cropping test site (G), CK, J, K and F treatments was 1339, and the number of unique bacterial OTU was 267, 278, 271, 376 and 336, respectively. Compared with CK treatment, K and F treatment can increase the unique OTU number of soil bacteria with continuous cropping *G. uralensis* (**Figure 1**).

The number of common fungal OTU between soil sample before planting *G. uralensis* in rotating test site (R), CK, J, K and F treatments was 105, and the number of unique fungal OTU was 233, 169, 132, 98 and 148, respectively. The number of common fungal OTU between soil sample before planting *G. uralensis* in continuous cropping test site (G), CK, J, K and F treatments was 76, and the number of unique fungal OTU was 141, 133, 92, 104 and 121, respectively. Compared with CK, J, K and F treatments significantly reduced the number of unique OTU of rotating and continuous cropping *G. uralensis* soil fungi (**Figure 2**).

### 3.2 Effect of microbial fertilizers on soil bacterial and fungal richness and diversity in *G. uralensis*

Based on the above OTU numbers, the bacterial and fungal diversity index (*Shannon* index) and the richness index (*Chao1* index) were calculated, and the results are shown in **Table 5**. For rotating *G. uralensis*, K and F treatment significantly increased the soil bacterial *Chao1* index by 6.75% and 11.69% compared with CK. J treatment significantly increased the soil bacterial *Shannon* index by 3.19% compared with CK, while K and F treatment had no significant effect on its bacterial *Shannon* index. For continuous cropping *G. uralensis*, the microbial fertilizers application did not significantly increase the soil bacterial *Chao1* index, but it had a significant promotion effect on the bacterial *Shannon* index. Among them, J, K and F treatments significantly increased their bacterial *Shannon* index by 10.31%, 7.22%, and 12.03% compared with CK, respectively.

**Table 5:** Richness and diversity index of soil bacterial and fungal in rotating and continuous cropping *G. uralensis* under different treatments.

Crops for rotation	Treatment	Bacteria		Fungi	
		*Chao1* index	*Shannon* index	*Chao1* index	*Shannon* index
Crop rotation	R	2781.67 ± 27.47b	6.29 ± 0.03a	287.33 ± 12.90a	3.56 ± 0.16a
	CK	2336.67 ± 19.85c	6.26 ± 0.04a	194.33 ± 11.22bc	3.51 ± 0.13a
	J	2356.33 ± 11.24c	6.46 ± 0.05a	258.00 ± 7.84abc	3.08 ± 0.17ab
	K	2494.33 ± 20.95bc	6.20 ± 0.04ab	226.33 ± 8.04abc	3.15 ± 0.08ab
	F	2609.33 ± 27.43bc	6.27 ± 0.02a	277.67 ± 9.14ab	3.57 ± 0.07a
Continuous cropping	G	2334.33 ± 30.99c	5.91 ± 0.03bc	227.67 ± 8.55abc	2.74 ± 0.13ab
	CK	3197.67 ± 42.50a	5.82 ± 0.05c	227.00 ± 6.51abc	2.42 ± 0.15b
	J	2787.67 ± 27.15b	6.42 ± 0.02a	213.00 ± 9.87abc	2.34 ± 0.12b
	K	2441.33 ± 24.38c	6.24 ± 0.05a	187.67 ± 5.16c	2.76 ± 0.08ab
	F	2327.67 ± 36.77c	6.53 ± 0.08a	224.33 ± 7.78abc	2.31 ± 0.04b

Fertilization can significantly reduce the soil fungal richness and diversity in rotating and continuous cropping *G. uralensis*. J, K and F treatment significantly reduced the rotating *G. uralensis* soil fungal *Chao1* index by 11.24%, 26.99%, and 3.61% compared with R treatment, respectively. The J and K treatment significantly reduced the *Shannon* index of rotating *G. uralensis* soil fungi by 15.58% and 13.02% compared with R treatment, and 13.96% and 11.43% were significantly lower than the CK treatment. The J and K treatments significantly reduced the *Chao1* index of continuous cropping *G. uralensis* soil fungi by 6.57% and 21.39% compared with CK, respectively. The J and F treatments significantly reduced the *Shannon* index of continuous cropping *G. uralensis* soil fungi by 3.42% and 4.76% compared with CK, respectively (**Table 5**).

### 3.3 Effect of microbial fertilizers on bacterial and fungal community composition in *G. uralensis* soil

#### 3.3.1 Soil bacterial community composition and their relative abundance changes

**Figure 3:**
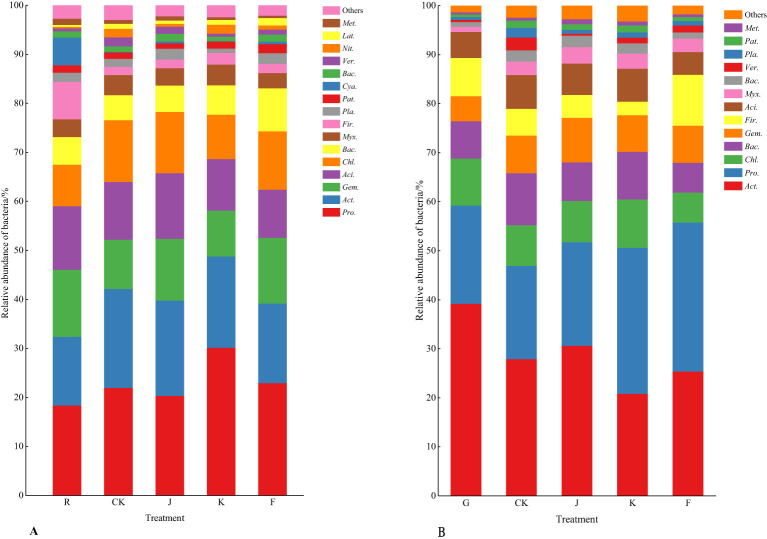
Composition of soil bacterial community in rotating **(A)** and continuous cropping **(B)** *G. uralensis* between different treatments (phylum level) Figure A: *Pro.*, *Proteobacteria*; *Act.*, *Actinobacteria*; *Gem.*, *Gemmatimonadota*; *Aci.*, *Acidobacteriota*; *Chl.*, *Chloroflexi*; *Bac.*, *Bacteroidota*; *Myx.*, *Myxococcota*; *Fir.*, *Firmicutes*; *Pla.*, *Planctomycetota*; *Pat.*, *Patescibacteria*; *Cya.*, *Cyanobacteria*; *Ver.*, *Verrucomicrobiota*; *Nit.*, *Nitrospirota*; *Lat.*, *Latescibacterota*. Others, Bacterial unclassified. Figure B: *Act.*, *Actinobacteria*; *Pro.*, *Proteobacteria*; *Chl.*, *Chloroflexi*; *Bac.*, *Bacteroidota*; *Gem.*, *Gemmatimonadota*; *Fir.*, *Firmicutes*; *Aci.*, *Acidobacteriota*; *Myx.*, *Myxococcota*; *Ver.*, *Verrucomicrobiota*; *Pla.*, *Planctomycetota*; *Pat.*, *Patescibacteria*. Others, Bacterial unclassified. The same as following.

**Figure 4:**
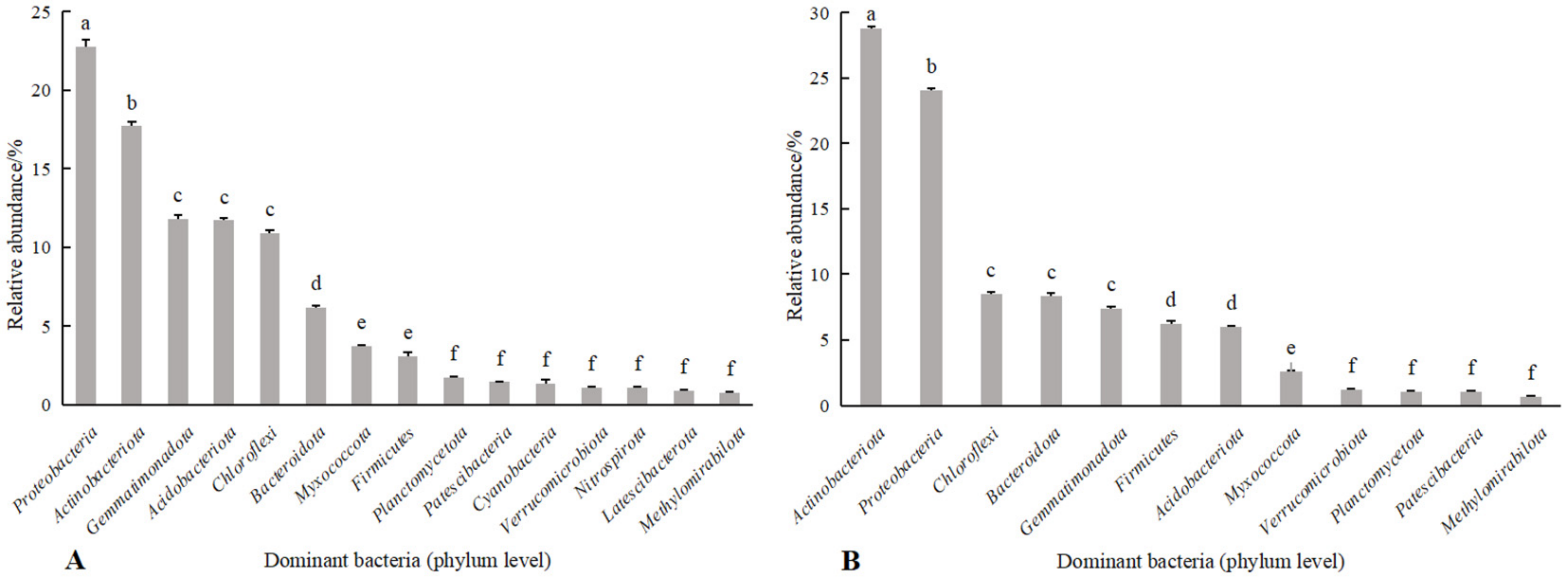
Relative abundance of soil bacterial communities (phylum level) in rotating **(A)** and continuous cropping **(B)** of *G. uralensis* Different lowercase letters indicate significant differences between different dominant bacterial communities (*P*<0.05). The same as following.

Combining **Figures 3**, **4** shows, rotating *G. uralensis* soil bacterial communities in 17 phyla. There were 6 classes of dominant bacterial communities with clear classification and relative abundance greater than 5.00%, namely *Proteobacteria*, *Actinobacteria*, *Gemmatimonadota*, *Acidobacteriota*, *Chloroflexi*, and *Bacteroidota* (**Figure 3A**). In continuous cropping *G. uralensis*, soil bacterial community in 14 phyla. There are 7 classes of dominant bacterial communities with clear classification and relative abundance greater than 5.00%, namely *Actinobacteria*, *Proteobacteria*, *Chloroflexi*, *Bacteroidota*, *Gemmatimonadota*, *Firmicutes*, and *Acidobacteriota*. In rotating and continuous cropping of *G. uralensis*, the relative abundance of dominant bacteria in soil *Actinobacteria* and *Proteobacteria* were significantly higher than those of other bacterial communities (**Figure 3B**).

**Figure 5:**
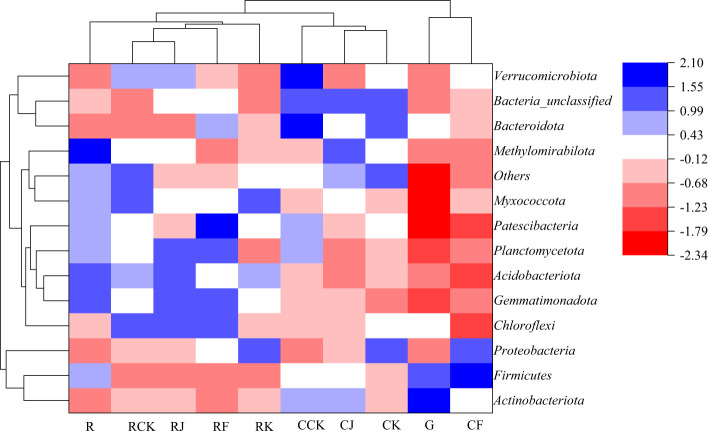
Clustering analysis of soil bacterial communities under different treatments (phylum level) R, Soil sample before planting *G. uralensis* in rotating cropping. RCK, Conventional fertilization control in rotating *G. uralensis*. RJ, *Bacillus amylolytica* in rotating *G. uralensis*. RK, *Bacillus subtilis* in rotating *G. uralensis*. RF, Complex microbial agent in rotating *G. uralensis*; G, Soil sample before planting *G. uralensis* in continuous cropping. CCK, Conventional fertilization control in continuous cropping *G. uralensis*. CJ, *Bacillus amylolyticus* in continuous cropping *G. uralensis*. CK, *Bacillus subtilis* in continuous cropping *G. uralensis*. CF, Complex microbial agent in continuous cropping *G. uralensis*. The same as following.

All treatments were clustered to analyze the similarities and differences in bacterial community composition between the different treatments, and the results are shown in **Figure 5**. The fertilization treatments of rotating *G. uralensis* RCK, RJ, RF and RK treatments were clustered into one class and R treatment was arranged separately. CJ, CK and CCK treatments were treated with continuous cropping *G. uralensis*, and the G treatment was arranged separately. It indicated that the bacterial community composition and its abundance of the soil before *G. uralensis* cultivation were significantly different from that after fertilization. In other words, both conventional fertilization and microbial fertilizers could change the composition and abundance of soil bacterial communities in rotating and continuous cropping *G. uralensis*.

The effects of different fertilization treatments on the relative abundance of major dominant bacteria in rotating and continuous cropping *G. uralensis* soils are shown in **Table 6**. Compared to the relative abundance of soil dominant bacteria before planting *G. uralensis* in rotation (R) and continuous cropping (G), the relative abundance of *Pro.*, *Act.*, and *Chl.* in rotating *G. uralensis* were significantly increased by 2.21~11.97, 2.03~6.33, and 0.97~5.30 percentage points under different fertilization treatments, and the relative abundance of *Pro.*, *Gem.*, and *Aci.* in continuous cropping *G. uralensis* increased significantly by 1.51~10.72,1.94~4.05 and 1.16~2.84 percentage points. Compared to the CK treatment with rotating *G. uralensis*, the J treatment significantly increased the relative abundance of *Gem.* and *Aci.* dominant bacteria by 3.22 and 2.88 percentage points, respectively. The K treatment significantly increased the relative abundance of *Pro.* dominant bacteria by 8.09 percentage points, and the F treatment significantly increased the relative abundance of *Gem.* and *Bac.* dominant bacteria by 3.11 and 3.85 percentage points, respectively. Compared to the CK treatment with continuous cropping *G. uralensis*, the J treatment significantly increased the relative abundance of *Pro.* and *Act.* dominant bacteria by 2.33 and 2.97 percentage points, respectively. The K treatment significantly increased the relative abundance of *Pro.* and *Chl.* dominant bacteria by 10.91 and 2.30 percentage points, respectively, and the F treatment significantly increased the relative abundance of *Pro.* dominant bacteria in soil by 11.54 percentage points.

**Table 6:** Effects of treatments on the relative abundance of soil dominant bacterial communities in rotating and continuous cropping *G. uralensis*.

Crops forrotation	Treatment	Bacterial dominant population (%)					
		*Pro.*	*Act.*	*Gem.*	*Aci.*	*Chl.*	*Bac.*
Crop rotation	R	18.24±0.21d	14.46±0.11d	14.25±0.11a	13.49±0.14ab	8.46±0.11b	6.13±0.11b
	CK	22.12±0.23b	20.79±0.22a	10.56±0.12b	12.69±0.12b	13.76±0.12a	5.42±0.10b
	J	20.45±0.30c	19.44±0.17b	13.78±0.14a	14.97±0.14a	13.53±0.14a	5.31±0.09b
	K	30.21±0.33a	19.13±0.21b	9.34±0.12bc	11.68±0.12c	9.43±0.12b	6.16±0.08b
	F	23.45±0.15b	16.49±0.13c	13.67±0.15a	10.43±0.14c	12.66±0.13a	9.27±0.07a
Continuous cropping	G	20.25±0.09b	39.37±014a	5.43±0.04c	5.33±0.05bc	10.79±0.08a	8.69±0.08b
	CK	19.43±0.11c	28.46±0.11c	8.76±0.07ab	7.72±0.06a	8.46±0.05b	11.73±0.07a
	J	21.76±0.13b	31.43±0.02b	8.27±0.06ab	6.49±0.04b	8.53±0.06b	8.86±0.06b
	K	30.34±0.14a	21.55±0.13e	7.37±0.05bc	7.88±0.05a	10.76±0.07a	10.34±0.07a
	F	30.97±0.12a	25.73±0.14d	9.48±0.06a	8.17±0.07a	6.49±0.06c	6.59±0.05c

#### 3.3.2 Soil fungal community composition and their relative abundance changes

**Figure 6:**
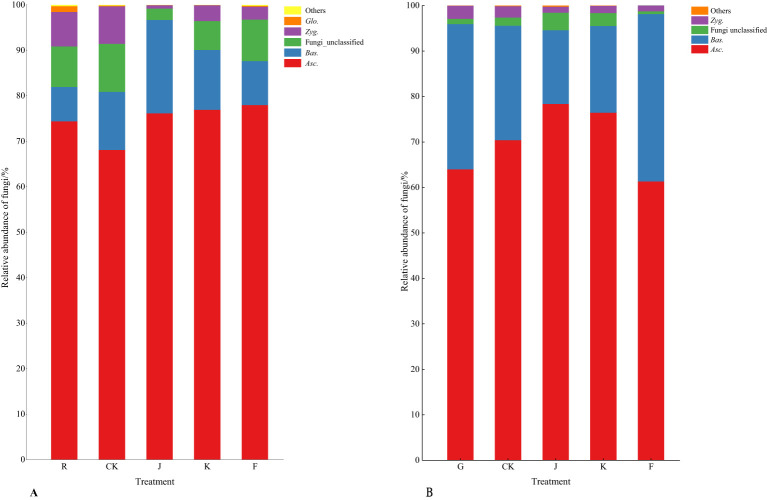
Composition of soil fungal community in rotating **(A)** and continuous cropping *G. uralensis* **(B)** between different treatments (phylum level) Figure A: *Asc.*, *Ascomycota*; *Bas.*, *Basidiomycota*; *Zyg.*, *Zygomycota*; *Glo.*, *Glomeromycota*. Others, Bacterial unclassified. Figure B: *Asc.*, *Ascomycota*; *Bas.*, *Basidiomycota*; *Zyg.*, *Zygomycota*. Others, Bacterial unclassified.

**Figure 7:**
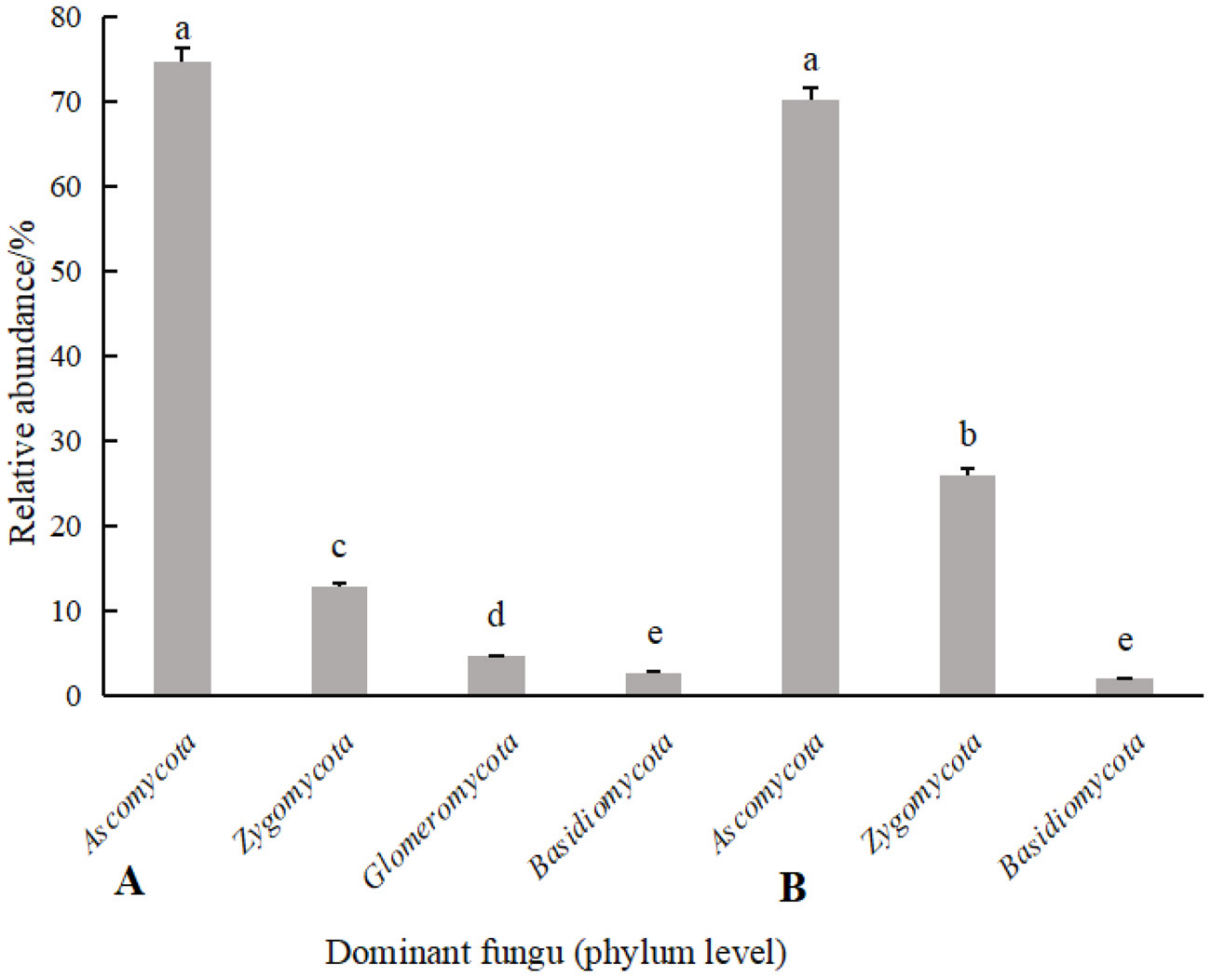
Relative abundance of soil fungal communities in rotating **(A)** and continuous cropping **(B)** of *G. uralensis* (phylum level).

According to **Figures 6**, **7**, the soil dominant fungal communities of rotating *G. uralensis* were *Ascomycota*, *Basidiomycota*, *Zygomycota*, and *Glomeromycota*, with relative abundance of 74.74%, 12.74%, 4.58%, and 0.32%. The dominant fungal community of continuous cropping *G. uralensis* was *Ascomycota*, *Basidiomycota* and *Zygomycota*, and the relative abundance was 70.12%, 25.85%, and 1.81% respectively. The relative abundance of soil *Basidiomycota* in continuous cropping *G. uralensis* was 13.11 percentage points higher than rotating *G. uralensis*.

**Figure 8:**
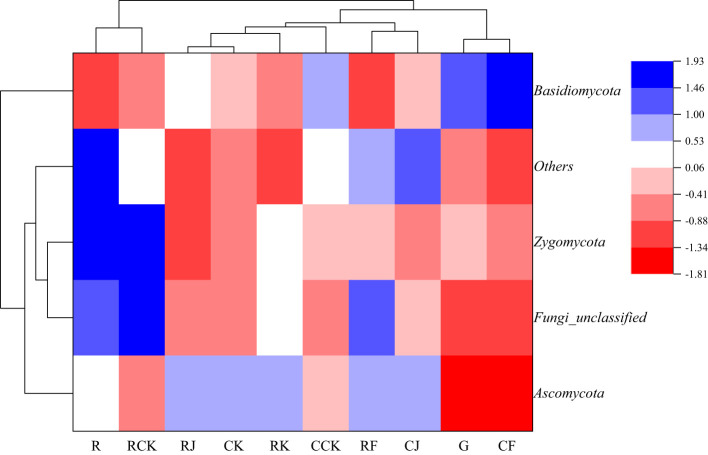
Clustering analysis of soil fungal communities under different treatments (phylum level).

The results of the similarity and differential clustering analysis of soil fungal community composition across all treatments are shown in **Figure 8**. Under different fertilization treatments, the relative abundance of *Ascomycota* and *Zygomycota* of rotating and continuous cropping *G. uralensis* was similar in each other, while *Basidiomycota* was quite different from the above two fungi, such as *Ascomycota* and *Zygomycota*. In addition, RJ, RK, and RF treatments clustered into a large class, R and RCK treatment alone clustered into one class. This indicated that the composition and abundance of soil fungal community of rotating *G. uralensis* were significantly different from that of conventional fertilization compared with microbial fertilizers application. The G treatment with other treatments separately into a class, such as CJ, CK, and CCK treatments, indicated that both conventional fertilization and microbial fertilizers could significantly affect the fungal community composition and abundance of continuous cropping *G. uralensis*. The RJ and RK treatments cluster into a class, simultaneously, the CK and CJ treatments also cluster into a class, suggested some similarity in the fungal community composition and abundance of rotating and continuous cropping *G. uralensis* soil treated by *Bacillus amyloliquefaciens* and *Bacillus subtilis*.

Compared to the relative abundance of soil dominant fungi before planting *G. uralensis* in rotation (R) and continuous cropping (G), microbial fertilizers were not significant in reducing the relative abundance of soil dominant fungal communities in rotating *G. uralensis*. However, microbial fertilizers could significantly reduce the relative abundance of the dominant fungal communities *Asc.*, *Bas.* and *Zyg.* of continuous cropping *G. uralensis* (**Table 7**). For the soil dominant fungi of rotating *G. uralensis*, compared with CK treatment, the F treatment significantly reduced the relative abundance of soil *Bas.* and *Zyg.* by 3.00 and 4.35 percentage points, while J and K treatment did not significantly reduce the effect. For soil dominant fungi of continuous cropping *G. uralensis*, compared with CK treatment, F treatment significantly reduced the relative abundance of *Asc.* by 8.93 percentage points, and the J and K treatment significantly reduced the relative abundance of *Bas.* by 9.04 and 5.68 percentage points, respectively. The J, K and F treatments significantly reduced the relative abundance of soil *Zyg.* by 2.24, 0.98 and 1.57 percentage points compared with CK treatment, respectively (**Table 7**).

**Table 7:** Effects of treatments on the relative abundance of dominant fungal communities in soil of rotating and continuous cropping *G. uralensis*.

Crops for rotation	Treatment	Fungal dominant population (%)		
		*Asc.*	*Bas.*	*Zyg.*
Crop rotation	R	74.61±0.08b	8.67±0.09d	8.76±0.04a
	CK	68.54±0.08c	13.16±0.14b	8.14±0.03a
	J	76.16±0.06ab	21.49±0.16a	7.46±0.03a
	K	77.49±0.08a	13.78±0.13b	3.47±0.02b
	F	78.94±0.02a	10.16±0.08c	3.78±0.01b
Continuous cropping	G	64.67±0.02c	32.43±0.13b	3.43±0.01a
	CK	70.29±0.05b	25.47±0.11c	3.76±0.02a
	J	78.94±0.02a	16.43±0.08e	1.46±0.01c
	K	76.57±0.06a	19.79±0.09d	2.78±0.01b
	F	61.46±0.07d	37.73±0.16a	1.86±0.01c

In conclusion, under the effects of microbial fertilizers, the composition of dominant bacterial and fungal communities in rotating and continuous cropping *G. uralensis* was basically the same, but the relative abundance of the dominant microflora varied significantly. Different microbial fertilizers could significantly increase the relative abundance of dominant bacterial communities of soil *Proteobacteria, Actinobacteria* and *Chloroflexi* in rotating and continuous cropping *G. uralensis*. In particular, microbial fertilizers could significantly reduce the relative abundance of dominant fungi in *Ascomycota*, *Basidiomycota* and *Zygomycota* of continuous cropping *G. uralensis*. Among them, the effect of the complex microbial agent was the most significant.

### 3.4 Effect of microbial fertilizers on soil bacterial and fungal community structure in *G. uralensis*

#### 3.4.1 Analysis of the differences in soil bacterial community structure

**Figure 9:**
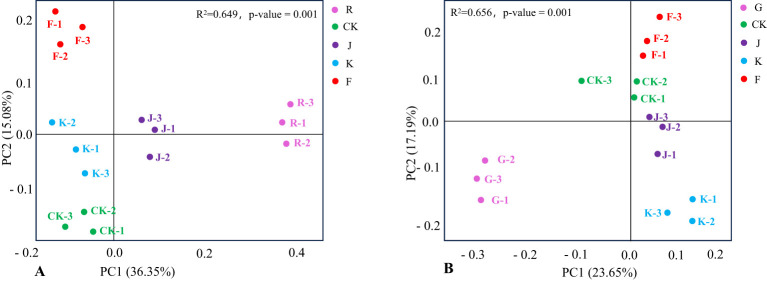
PCoA of soil bacterial communities in rotating **(A)** and continuous cropping **(B)** *G. uralensis* under different treatments.

Further performed PCoA (Principal Coordinates Analysis) for all soil samples, thus reflected the differences in the distribution of soil bacterial and fungal community structure between the different treatments, and tested by permutational multivariate analysis of variance whether the difference in soil bacterial and fungal community structure was significant under different fertilization treatments. The closer the distance between the samples, the higher the structural similarity of species composition. **Figure 9A** shows that the interpretation rate of PC1 was 36.35% and PC2 was 15.08%, and the permutational multivariate analysis *R^2^* = 0.649, *P* = 0.001, indicated that the structure of soil bacterial communities in rotating and continuous cropping *G. uralensis* were significantly different under different fertilization treatments. For the rotating of *G. uralensis*, the close distance between CK, K, and J treatments indicated that the structure of soil bacterial community composition was similar between conventional fertilization treatments and *Bacillus amyloliquefaciens* and *Bacillus subtilis*. The F treatment is distant from J and K treatments indicated a significant difference in the spatial structure distribution of soil bacterial community composition between the F treatment and the J and K treatments. Among them, the bacterial community composition of complex microbial agent treatment was abundant and diverse in spatial distribution compared with other treatments.

Under different treatments of continuous cropping *G. uralensis*, the G treatment is distant from CK, J, F and K treatments, and the CK, J, and F treatments were located closer to each other. It showed that both conventional fertilization and microbial fertilizers coould significantly affect the bacterial community composition structure of continuous cropping *G. uralensis* soil. In this, the structure of soil bacterial community composition of *Bacillus amyloliquefaciens* and complex microbial agent treatment was basically similar to that of conventional fertilization treatment. Furthermore, the K treatment is distant from J and F treatment, suggested that the composition of soil bacterial community under the treatment of *Bacillus subtilis* was significantly different from that under the treatment of *Bacillus amyloliquefaciens* and complex microbial agent (**Figure 9B**).

#### 3.4.2 Analysis of the differences in soil fungal community structure

**Figure 10:**
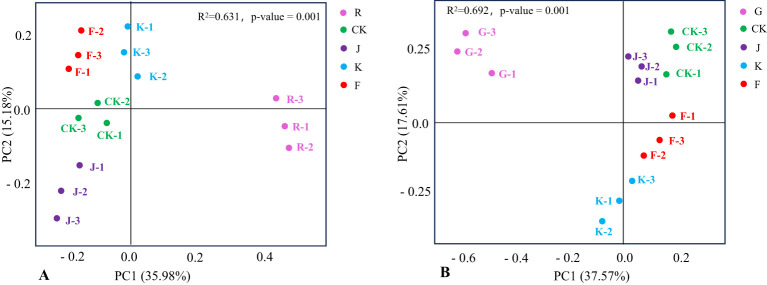
PCoA of soil fungal communities in rotating **(A)** and continuous cropping **(B)** *G. uralensis* under different treatments.

The R and G treatments of rotating and continuous cropping *G. uralensis* were far from other fertilization treatments, indicated that fertilization significantly affected the fungal community structure in rotating (*R^2^* = 0.631, *P* = 0.001) and continuous cropping (*R^2^* = 0.692, *P* = 0.001) *G. uralensis*. Moreover, the distance between K and F treatment of rotating and continuous cropping *G. uralensis* was close, and the distance between K and J treatment was relatively distant. It suggested that the soil fungal community structure of *Bacillus subtilis* treatment and complex microbial agent treatment in rotating and continuous cropping *G. uralensis* were similar, but there are differences between them and the treatment of *Bacillus amyloliquefaciens* (**Figure 10**).

### 3.5 Effect of microbial fertilizers on soil microbial KEGG metabolic pathways in *G. uralensis*

**Figure 11:**
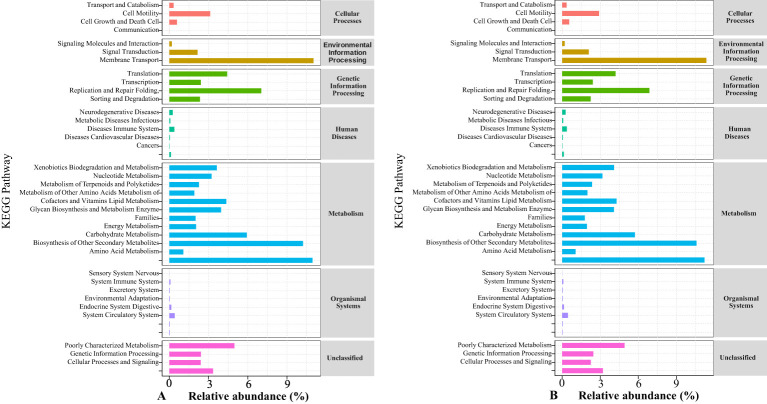
Annotated pathway statistical map of soil bacterial community gene sequences of rotating **(A)** and continuous cropping **(B)** *G. uralensis* in KEGG database.

Changes in soil bacterial and fungal richness, diversity, community composition, and structural distribution can cause changes in soil microbial metabolic functions. Therefore, in this study, the sequence of bacterial community genes in rotating and continuous cropping *G. uralensis* were further aligned with KEGG (Kyoto Encyclopedia of Genes and Genomes) data, and later, functional annotation was performed. The results are shown in **Figure 11**. Under the action of different microbial fertilizers, the main functions of rotating and continuous cropping *G. uralensis* soil bacterial functional genes in the primary metabolic pathway of the KEGG database included metabolism, genetic information processing and eavironmental information processing. Among them, the metabolism accounted for the highest proportion, followed by genetic information processing, this showed that the function of soil microorganisms in rotating and continuous cropping *G. uralensis* is mainly by metabolism. In the secondary metabolic pathway of the KEGG database, the main functions of rotating and continuous cropping *G. uralensis* soil microorganisms are carbohydrate metabolism, amino acid metabolism and energy metabolism. There was no significant difference in the proportion of main functions of soil microorganisms between rotating and continuous cropping *G. uralensis*. Suggested that under the action of the microbial fertilizers, the change of continuous cropping and rotating system had little effect on the main functions of *G. uralensis* soil microorganisms (**Figure 11**).

## 4 Discussion

In this study, under different fertilization treatments, the species and quantity of soil bacterial and fungal communities in rotating *G. uralensis* were higher than those in continuous cropping *G. uralensis* (**Table 5**). This result was analyzed according to the research results of “the soil microbial richness and diversity of rotaing *Zea mays* L. were significantly higher than that of continuous stubble (Ma et al., 2024; Gillespie et al., 2021)”. On the one hand, because microbial fertilizers could significantly enrich the species and number of microorganisms in the *G. uralensis* surface soil (Wen et al., 2024). On the other hand, the root system of rotating *G. uralensis* is deeper than that of continuous cropping *G. uralensis*, and the deep *G. uralensis* root system is more conducive to activating the microbial life activities in deep soil. This “top-down” mechanism of action, the common activation of soil microbial life activities, so that the rotating *G. uralensis* soil microbial species and quantity increase faster (Anton et al., 2023; Giuseppe et al., 2021; Roohi et al., 2020).

The results of this study showed that *Bacillus amylodiolitica*, *Bacillus subtilis* and complex microbial agent significantly increased the soil bacterial diversity of continuous cropping *G. uralensis* by 7.22% to 12.03%, and also significantly reduced the soil fungal richness and diversity of continuous cropping *G. uralensis* by 4.76% to 20.96%. However, the effect of microbial fertilizers on soil bacteria and fungi of rotating *G. uralensis* was not particularly significant, compared with the effect of microbial fertilizers on continuous cropping *G. uralensis* (**Tables 5**–**7**). Analysis of its reasons, may be that the original microorganisms in the soil of rotating *G. uralensis* had many kinds, high abundance and strong adaptability, preferentially occupied the nutrient or reproductive sites, and inhibited the growth and reproduction of functional microorganisms in the microbial fertilizers (Lijing et al., 2024; Rose et al., 2024). For the soil of continuous cropping *G. uralensis*, the species and number of original microorganisms are small and the ability to compete for breeding sites is weak, while the functional microorganisms in microbial fertilizers are abundant and have strong enrichment ability, which can preferentially propagat in the soil of continuous cropping *G. uralensis* (He et al., 2024; Li et al., 2024). In addition, after the application of microbial fertilizers for continuous cropping *G. uralensis*, the soil nutrient content increases, and the soil environment is more conducive to the growth of beneficial microflora in microbial fertilizers (Holatko et al., 2024). Therefore, in the results of this experiment, the effect of microbial fertilizers affected the soil microbial community structure of continuous cropping *G. uralensis* was significantly stronger than that of rotating *G. uralensis*. In addition to that, the microorganisms in microbial fertilizers grow and play its role, itself requires a certain adaptation time, and the overall regulation of soil microbes, it also takes a long time (Han et al., 2024; Xin et al., 2023). This may also be the main reason for the small effect of microbial fertilizers on the soil microbes of rotating *G. uralensis* in a short time.

In this reserach, the effect of the complex microbial agent on the species and quantity of *G. uralensis* soil was most remarkable, which increased the relative abundance of *Gemmatimonadota* and *Proteobacteria* of rotating and continuous cropping *G. uralensis* soil by 0.03 and 0.11 percentage points, respectively, and significantly reduced the relative abundance of dominant fungi in *Basidiomycota* and *Zygomycota* was 0.09 and 0.02 percentage points, respectively (**Tables 6**, **7**). This result was consistent with the results of the study that “the compound bacterial fertilizer had the best effect on regulating the soil microflora structure of *Lilium brownii* var. and *Pseudostellaria heterophylla* in continuous cultivation (Yan et al., 2022; Lin et al., 2012)”. The main reason is that the complex microbial agent itself contains a complete variety of functional microorganisms with a stable structure, and the synergistic ability of different strains is stronger than that of single strain type microbial fertilizers (Vitorino et al., 2024; Lixinyue et al., 2024).

*Basidiomycota* is a rotten soil fungal pathogen, which occupies a relatively high proportion in soil fungi, and it is easy to enrich and develop into dominant fungal communities with the increase of plant continuous cropping years (Manici et al., 2020; Traxler et al., 2023). Consequently, leading to the aggravation of soil “fungalization” and the increased incidence of plant root rot (Sang et al., 2024). In the present study, the relative abundance of *Basidiomycota* in continuous cropping *G. uralensis* soil was significantly higher by 0.12 percentage points than that of rotating *G. uralensis* (**Figures 4**, **7**), which is consistent with the above phenomenon.

Soil microorganisms decompose and transform soil nutrients through multiple metabolic pathways (Liu et al., 2024; Lihualiang et al., 2024). Metabolism, the main pathway by which *Astragalus memeranaceus* soil microorganisms exert their effects. Moreover, this metabolic pathway accounted for more than 50.09% of the different fertilization treatments of crop rotation and continuous cropping of *Astragalus memeranaceus* (Qin et al., 2024). The results of this study also indicated that the microbial activity of rotating and continuous cropping *G. uralensis* is dominated by metabolism under the action of different microbial fertilizers (**Figure 11**). Uniformly indicated that the metabolic capacity of soil microorganisms is enhanced, which is conducive to improving the decomposition efficiency of soil organic matter and the absorption efficiency of soil nutrients by plants, and ensuring the supply of essential nutrients for plant growth.

## 5 Conclusion

Continuous cropping would lead to the decrease of the richness and diversity of *G. uralensis* soil microorganisms, and the serious “fungalization” of soil. After the application of microbial fertilizers such as *Bacillus amyloliquefaciens*, *Bacillus subtilis*, and complex microbial agent, the richness and diversity of continuous cropping *G. uralensis* soil bacteria were significantly increased (by 7.22% to 12.03%), and the fungal richness and diversity were significantly decreased (by 3.42% to 4.76%), and the soil microbial community structure was optimized. These microbial fertilizers were better in optimizing soil microbial community structure of continuous cropping *G. uralensis* than in rotating *G. uralensis*. This provided theoretical support for the efficient fertilization of *G. uralensis* cultivation in the desert and semi-desert areas of western China, in particular, the complex microbial agent was very promising in *G. uralensis* cultivation. However, there are still some shortcomings in this study: “linking soil microorganisms, soil physical and chemical properties and *G. uralensis* yield and quality, and analyzing their interrelationship and mechanism of action”. In future studies, should combine equation models, metabolomics and transcriptomics, focusing on the mechanism of action between soil microbiotic environment and *G. uralensis* yield and quality, and explore the influence of different strains in microbial fertilizers on the yield and quality of *G. uralensis*.

## Data Availability

The original contributions presented in the study are publicly available. This data can be found here: https:/www.ncbi.nlm.nih.gov/bioproject/PRJNA1200788.
